# Epidemiology of COVID-19 and the Utility of Cycle Threshold (Ct) Values in Predicting the Severity of Disease

**DOI:** 10.7759/cureus.43679

**Published:** 2023-08-18

**Authors:** Anuja George, Thamizharasi Murugan, Srinivasan Sampath, Madhusudhan N S

**Affiliations:** 1 Microbiology, Indira Gandhi Medical College & Research Institute, Puducherry, IND; 2 Microbiology, Sri Venkateshwaraa Medical College Hospital and Research Centre, Puducherry, IND

**Keywords:** south india, polymerase chain reaction (pcr), viral load, covid-19, coronavirus disease 2019, cycle threshold, sars-cov-2

## Abstract

Objectives: Advanced molecular diagnostic methods like real-time polymerase chain reaction (PCR) play a vital role in the early recognition of viral infections, including the coronavirus disease 2019 (COVID-19). Therefore, in the context of the recent COVID-19 pandemic, this study aimed to determine the correlation of cycle threshold (Ct) values with symptoms in COVID-19-positive patients.

Materials and methods: A retrospective study was conducted in a virus research diagnostic laboratory (VRDL) at a COVID-19-dedicated tertiary care hospital in South India. A total of 5563 COVID-19-positive patients were analyzed for symptom spectrum and duration of illness with Ct values of severe acute respiratory syndrome coronavirus 2 (SARS-CoV-2).

Results: Around 80% (n= 4401) of the patients were symptomatic and the rest were asymptomatic. Among the symptomatic patients, fever (66%) was the most common symptom. About 44% of symptomatic patients had a low Ct value (Ct ≤ 24). There was a significant difference in symptoms among patients with low, medium, and high Ct values. In the subpopulation of symptomatic patients analyzed for the association of Ct value and duration of illness, the mean duration of illness was three days and almost 88% of the patients were tested within five days of onset of symptoms. It has been observed that a shorter duration of illness lowers the Ct values. A significant association was seen between the mean Ct value and days since symptom onset (p-value = 0.016).

Conclusion: Most of the symptomatic patients had lower Ct values in comparison to the asymptomatic patients. A significant association between low Ct values and the duration of symptoms observed in our study explains the viral dynamics, i.e., higher viral shedding at the onset of symptoms and declines thereafter.

## Introduction

In recent years we witnessed the emergence and re-emergence of many diseases, causing epidemics and pandemics [1]. These are still a matter of particular concern as they resulted in substantial morbidity and mortality. Even though many are recognized infectious diseases, significantly greater numbers remain unrecognized [2]. All these infectious diseases cause significant healthcare expenditure, especially in resource-poor countries like India [1]. In addition to accurate pathogen recognition, outcomes from infectious diseases also correlate with the time to pathogen identification [2,3]. Various epidemics and pandemics caused by the Ebola virus, influenza virus, Zika virus, and coronavirus disease 2019 (COVID-19) in the recent past highlighted the necessity of early detection and the role of molecular diagnostics in identification.

Among the molecular methods, polymerase chain reaction (PCR) is a powerful tool that can detect even very small amounts of nucleic acids. Because of the exponential amplification of the specified sequence, it has higher sensitivity [3]. Thus the establishment of PCR-based diagnostic methods enabled quick, reliable, and accurate detection of infecting pathogens [2]. Quantification of target sequences by real-time quantitative PCR (qPCR) involves continuous analysis of variation in the number of fluorescence signals during the amplification reaction. When compared to conventional PCR where data is analyzed at the end of PCR, the real-time PCR technology does a real-time detection of the number of amplicons generated in each amplification cycle. Thus, this eliminated the requirement for post-amplification analysis of the samples and paved the way for fully automated detection systems [3].

Optimized qPCR tests showed very high sensitivity, with limits of detection of 1-10 targeted molecules per reaction. Due to these properties, qPCR has become an important tool for the detection and monitoring of viral infectious diseases. However, nowadays commercial qPCR assays are available only for a smaller number of viral pathogens; for example, cytomegalovirus (CMV), hepatitis viruses B and C (HBV, HCV), human immunodeficiency virus (HIV-1), human papillomavirus (HPV), and severe acute respiratory syndrome (SARS)-associated coronavirus (CoV). But many other in-house methods of qPCR assays have been developed for other viral targets and were implemented in clinical diagnosis [3].

Since the end of 2019, millions of people have died worldwide because of COVID-19 caused by severe acute respiratory syndrome coronavirus 2 (SARS-CoV-2) [4]. In a short time period, India became one of the epicentres of this pandemic globally, accounting for one-sixth of all cases that were reported [5]. Patients with SARS-CoV-2 exhibited a varying degree of disease severity, ranging from a lack of symptoms to the need for intensive care, which can result in fatal outcomes [6]. Quantitative reverse transcription-PCR (qRT-PCR) is considered the gold standard laboratory technique for identifying SARS-CoV-2. This method can identify viral RNA during the initial days of symptom onset, during the early stages of the disease, or even during pre-symptomatic or post-symptomatic phases. Upper respiratory specimens, such as nasopharyngeal or oropharyngeal swabs, aspirates or washes, sputum, and bronchoalveolar fluids, are used most frequently for SARS-CoV-2 qRT-PCR testing [7].

Many molecular qRT-PCR kits have been approved by the United States Food and Drug Administration (FDA) and are widely accessible for the detection and amplification of SARS-CoV-2 RNA in addition to tests advised by the WHO [8]. For the detection of SARS-CoV-2, several viral target genes have been employed, including the spike (S), nucleocapsid (N), RNA-dependent RNA polymerase (RdRp), open reading frame (ORF) 1, and envelope (E) genes [9]. In real-time PCR assays, absolute copy numbers of the target of interest can be assessed by the appropriate calibration of assays and the use of standard curves. Results are displayed as amplification plots using a series of fluorescence signal measurements taken at particular time points during the process of amplification [3].

Results of COVID-19 qRT-PCR tests are often qualitatively reported as positive or negative using a set cut-off based on the cycle threshold (Ct) value. The number of cycles needed for the fluorescence signal to cross the background threshold is known as the Ct value. Lower Ct values indicate a higher viral load since they are inversely related to viral load and can be used as an indirect technique for measuring viral RNA in samples [6]. Few review articles have found that the viral load peaks at/before the onset of symptoms and declines thereafter [10]. But it is still unknown whether RT-PCR Ct values have any role in understanding the symptom spectrum. With this background, in the context of the recent COVID-19 pandemic, this study aimed to determine the correlation of Ct values and symptoms among COVID-19-positive patients.

## Materials and methods

**Figure 1:**
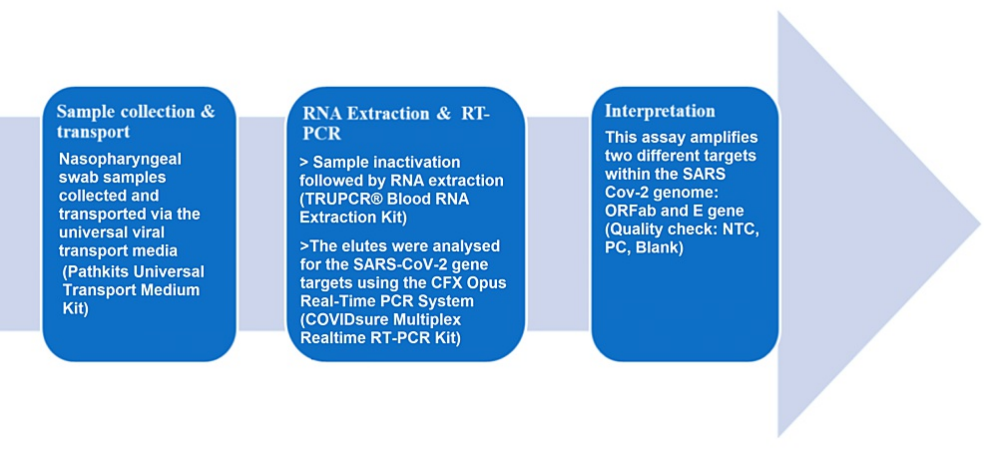
RT- PCR test procedure NTC: no template control; PC: positive control; RT-PCR: reverse transcription-polymerase chain reaction; SARS-CoV-2: severe acute respiratory syndrome coronavirus 2 Manufacturer details: Pathkits Universal Transport Medium Kit: Pathkits Healthcare Pvt Ltd, Gurugram, Haryana, India; TRUPCR® Blood RNA Extraction Kit: 3B BlackBio Biotech India Limited, Bhopal, Madhya Pradesh; CFX96 Real-Time PCR System: Bio-Rad Laboratories, Inc., Hercules, California, United States; COVIDsure Multiplex Realtime RT-PCR Kit: Trivitron Healthcare Pvt. Ltd, Chennai, India

The study was conducted in the Virus Research Diagnostic Laboratory (VRDL) under the Department of Microbiology, Indira Gandhi Medical College and Research Institute (IGMC&RI), Puducherry, India. Samples from various localities of Puducherry and Tamil Nadu as well as from suspected COVID-19 cases presenting to our hospital were received in the VRDL for COVID-19 testing during the pandemic. The qRT-PCR test procedure is depicted in Figure 1.

A retrospective study was done to analyze the Ct values of COVID-19-positive samples reported from May 2020 to December 2020 from the VRDL. Ethical clearance for the study was given by the Institute Ethics Committee of Indira Gandhi Medical College & Research Institute (reference number: 341/IEC-32/IGMC&RI/PP-21/2021 dated August 3, 2021). A total of 5563 COVID-19-positive patients were included for analysis in this study. Ct values were compared with respect to different age groups as well as clinical symptoms. Ct values of ORF1ab, which is the confirmatory gene in the kit used (TRUPCR® Blood RNA Extraction Kit, 3B BlackBio Biotech India Limited, Bhopal, Madhya Pradesh), were taken into consideration for comparison.

Ct values below 25, 25 to 30, and above 30 were categorized as high, moderate, and low viral load, respectively. Patients were categorized age-wise into <18 years (Pediatrics), 18-40 years (adults), 41- 60 years (Elderly) and >60 years (Old age). The patients were identified as symptomatic and asymptomatic based on history and data available from the specimen referral form (SRF). In addition, a subpopulation of symptomatic cases (239 COVID-19 patients) was analyzed for correlation of Ct value and duration of symptoms at the time of testing.

The data were analyzed using IBM SPSS Statistics for Windows, Version 20.0 (2011; IBM Corp., Armonk, New York, United States) and Microsoft Excel (Microsoft Office 2016; Microsoft Corporation, Redmond, Washington, United States). Continuous variables were expressed as mean and standard deviation and categorical variables were expressed as frequency and percentages. The association between the variables was determined using the chi-square test. A p-value of < 0.05 was considered statistically significant.

## Results

Of the total 5563 COVID-19-positive patients included in the study, the majority of them were males (n= 3380, 60.7%) as opposed to females (n= 2183, 39.3%). The age of the study population ranged from one year to 95 years with a median age of 40 years. The majority of the cases investigated were in the age range of 18-40 years (n = 2508, 45%), followed by the elderly age group of 41-60 years (n = 2269, 41%), with paediatric and old age populations being least impacted, as seen in Table 1.

**Table 1:** Age distribution of the COVID-19 patients COVID-19: coronavirus disease 2019

Age category	No. of patients (n= 5563)	Percentage (%)
Pediatric (≤17 years)	395	7%
Adults (18-40 years)	2508	45%
Elderly (41-60 years)	2269	41%
Old Age (≥ 61 years)	391	7%

**Figure 2:**
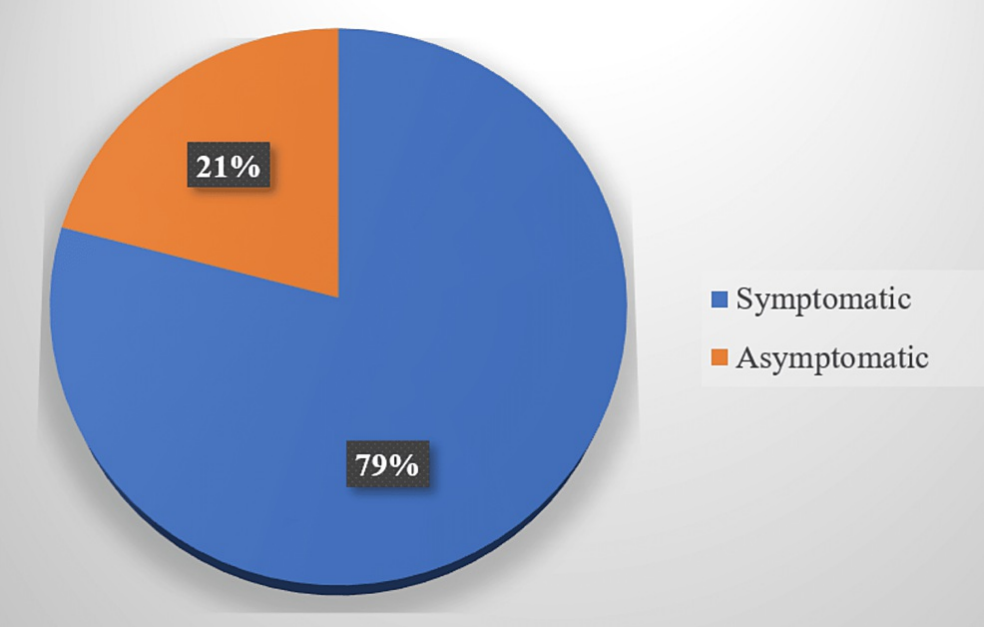
Distribution of symptom status among COVID-19 cases COVID-19: coronavirus disease 2019

Of the total cases analyzed, around 79% (n= 4401) were symptomatic and 21% (n= 1162) were asymptomatic (Figure 2).

**Figure 3:**
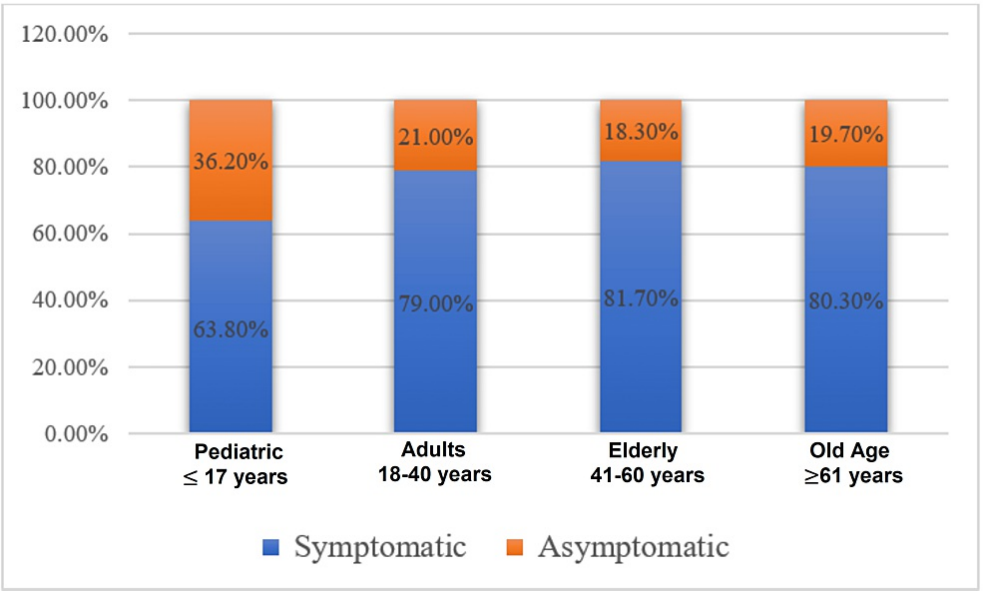
Symptom status in different age categories

All age groups had an asymptomatic proportion of around 20%, with the exception of paediatrics, where the proportion of asymptomatic patients was seen increased to 36% (Figure 3).

**Figure 4:**
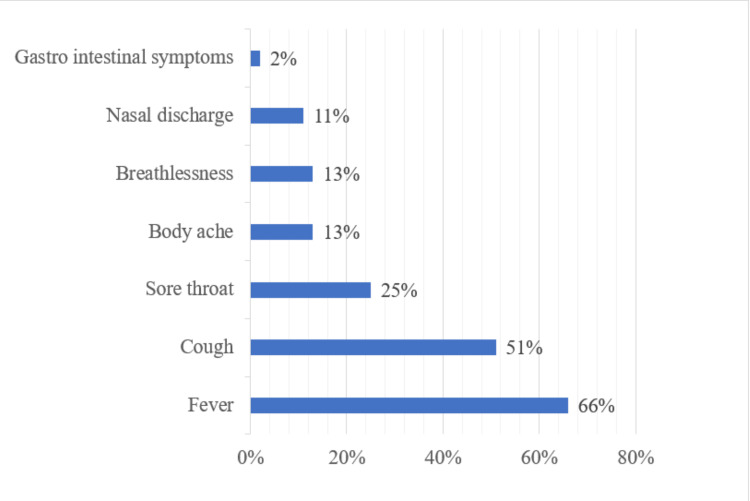
Spectrum of clinical symptoms among symptomatic patients

Among all symptomatic COVID-19 patients (n = 4401) included in the study, fever (66%) was the most prevalent symptom followed by cough (51%), and sore throat (25%). Nasal discharge and gastrointestinal symptoms were less frequent symptoms as shown in Figure 4.

For Ct value correlation, the study population were categorized into low Ct value (≤24), medium Ct value (25-30), and high Ct value (>30) groups [11], and these Ct value groups were compared to gender and age categories. It was found that there was no difference in Ct value with respect to gender (p-value: 0.8431). Compared among the age groups, the majority (44%) of the old age population were in the medium Ct value category in contrast to other age groups where the majority (~ 41%) were in the low Ct value category. There was a significant association between Ct value and age categories (p-value: 0.006) as summarized in Table 2.

**Table 2:** Distribution of Ct value with regard to gender and age Ct: cycle threshold

Characteristics	Category	Low Ct value (≤24)	Medium Ct value (25-30)	High Ct value (>30)	Total (n = 5563)	chi-square value	p-value
Gender	Female, n	909	850	424	2183	0.3413	0.8431
	%	42%	39%	19%			
	Male, n	1434	1296	650	3380		
	%	43%	38%	19%			
Age	Pediatric (≤17 years), n	160	147	88	395	17.98	0.006
	%	41%	37%	22%			
	Adults (18-40 years), n	1111	955	442	2508		
	%	44%	38%	18%			
	Elderly (41-60 years), n	929	871	469	2269		
	%	41%	38%	21%			
	Old Age (≥61 years), n	143	173	75	391		
	%	37%	44%	19%			

Furthermore, the Ct value categories were compared with symptom status and symptom spectrum (Table 3). While analyzing, it was noted that almost 45% (1915/4401) of the COVID-19 patients who presented with symptoms were having low Ct values (i.e., high viral load) and only 18% (792/4401) of them were in the high Ct category (i.e., low viral load). On the contrary, the asymptomatic patients had Ct values of varied distribution between medium (39%) and low Ct (37%) values. There is a statistical significance of symptom status and Ct value (p-value < 0.00001). It was also noted that the majority of patients who presented with breathlessness had medium (46%) and low (39%) Ct values.

**Table 3:** Ct value comparison with symptom status and symptom spectrum Ct: cycle threshold

Characteristics	Category	Low Ct Value (≤24)	Medium Ct Value (25-30)	High Ct Value (>30)	Total	chi-square value	p-value
Symptom status	Asymptomatic, n	428	452	282	1162	28.51	< 0.00001
	%	37%	39%	24%			
	Symptomatic, n	1915	1694	792	4401		
	%	44 %	38%	18%			
Symptom spectrum	Fever, n	1333	1113	462	2908	72.8	<0.0000001
	%	46%	38%	16%			
	Cough, n	946	890	424	2260		
	%	42%	39%	19%			
	Sore throat, n	527	391	192	1110		
	%	48%	35%	17%			
	Body ache, n	272	211	94	577		
	%	47%	37%	16%			
	Breathlessness, n	162	254	138	554		
	%	29%	46%	25%			
	Nasal discharge, n	214	174	86	474		
	%	45%	37%	18%			

**Figure 5:**
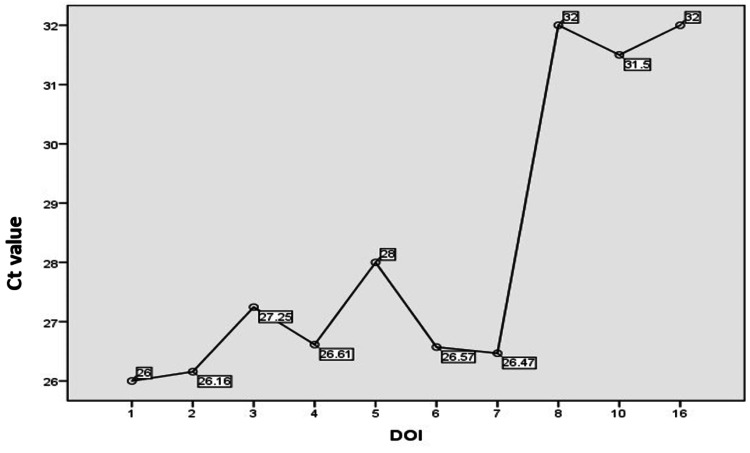
Duration of illness Vs mean Ct value DOI: duration of illness; Ct: cycle threshold

Among symptomatic patients, a subpopulation of 239 cases was analyzed for correlation of Ct value with days since symptom onset. The mean duration of illness was three days and almost 88% of the patients (211/239) were tested within five days of the onset of symptoms. It was found that there is a significant association (p-value is 0.016) between the mean Ct value and days since the onset of symptoms (Figure 5).

## Discussion

A total of 5563 COVID-19-positive patients were included in our study for analysis. Data were analyzed for demographic characteristics like gender and age. During analysis, more males (60.7%) were found to be positive than females and some studies from India also had similar male predominance [12,13]. The majority of the positive patients (45%) were adults (18-40 years), followed by the elderly (41%), with pediatric and old age populations being the least impacted.

A significant proportion of COVID-19-positive patients in our study were symptomatic (80%), whereas only 20% of patients were asymptomatic. These findings were consistent with reports by Li et al. (29.4% in 2020) [14] and El-Ghitany et al. (34.9% in 2021) [15], which showed a lower proportion of asymptomatic cases. However, these findings were contrary to the observations from many other Indian studies which reported an outranged prevalence of asymptomatic cases, including a hospital-based study by Soni et al. (2020) that reported 57.8 % asymptomatic cases [12], and a study by Krishnasamy et al. that reported 48.5% in 2020 from Chennai [16]. The presence of a significant asymptomatic population might help in efficiently spreading the infection without being caught by the tests or inviting medical attention.

As discussed above, the proportion of asymptomatic cases in our study was lower as compared to some other Indian reports [12,16] but the pediatric population had a slightly higher percentage (36%) of asymptomatic cases in comparison to all other age groups (20%). This observation of a higher proportion of asymptomatic infection among children was consistent with the reports from Du et al. (57.1% in 2020) [17]. A study by Qiu et al. (2020) also reported mild clinical features in about 47% of COVID-19-positive children, with asymptomatic presentation in around 30% of cases [18].

Fever and cough were the most commonly observed clinical presentation among the symptomatic patients. These findings were similar to the reports from various Indian studies like those from Soni et al. and Krishnasamy et al. [12,16]. However, in the current study, an overall predominance of fever (66%) cases was seen, which is contrary to some study reports where they observed cough as the predominant symptom [13].

Ct value is the number of PCR cycles at which the fluorescence signal crosses the threshold value, thus labelling a particular sample as positive or negative. Ct values are a surrogate marker of viral load and are inversely proportional to it. A lower Ct value signifies a higher viral load and vice versa [6]. As part of the study, the relation of Ct value with various demographic characteristics was analyzed.

In our study, we didn’t notice any difference in Ct value with respect to gender. Similarly, the absence of correlation between Ct value and gender was also noted in a multicenter cross-sectional study from other countries [19]. In our study, the majority of pediatric, adult (18-40 years), and elderly (40-60 years) populations showed low Ct value whereas the old age category showed mainly median Ct value. But in a retrospective study by Mishra et al., a significant percentage of adults (18-60 years) and elderly (>60 years) showed low Ct value (Ct value <25) compared to the young population [20].

In this study, an association of symptomatic presentation with respect to lower Ct values was noted and a similar finding was reported in a study in 2020 by Abdulrahman et al. [19]. This signifies that symptomatic individuals were found to harbor more viruses. This is also in corroboration with the studies by McEllistrem et al. [21] and Strutner et al. [22] in 2020 wherein symptomatic individuals were found to have higher viral load.

Among symptomatic cases, 239 cases were analyzed for correlation of Ct value with days since symptom onset. In our study, low Ct value (i.e., high viral load) was detected at or soon after the onset of symptoms and as day passes viral load gradually decreases with increasing Ct values; these findings were in accordance with other study reports [23]. Same time some other studies suggest that the interpretation of high Ct value results needs to be done in the context of the clinical situation and timing of testing relative to symptoms or exposure [24].

The major limitation of the present study is that it was a retrospective single-center study. Furthermore, patient follow-up was not done and hence relation to other patient factors such as comorbidities and vaccination were not assessed.

## Conclusions

Our study indicates that most of the COVID-19 cases were symptomatic and there is a higher proportion of asymptomatic cases seen in pediatrics. A significantly low Ct values (high viral load) observed in symptomatic cases, plays a considerable role in SARS-CoV-2 transmission. Except in the old age population, the majority of the symptomatic cases showed low Ct value. It was also observed that low Ct values were detected mainly in those who presented within seven days of symptom onset. As Ct values are influenced by various other factors, utilization of this alone as a viral load marker in clinical decision-making for any viral infectious diseases may not be a trustworthy strategy, especially in cases with high Ct values. A significant association between low Ct value and duration of symptoms observed in our study explains the viral dynamics, i.e., higher viral shedding on the onset of symptoms and declines thereafter. Hence it was evident that in case of any viral infectious diseases, testing and isolation at the onset of symptoms help to contain the pandemic and therefore providing the Ct values along with a positive report would help in better patient management.
